# ONLINE FORECASTING OF COVID-19 CASES IN NIGERIA USING LIMITED DATA

**DOI:** 10.1016/j.dib.2020.105683

**Published:** 2020-05-08

**Authors:** Kabir Abdulmajeed, Monsuru Adeleke, Labode Popoola

**Affiliations:** a Georgia Institute of Technology, Atlanta, GA, USA; b Osun State University, Osogbo, Nigeria

**Keywords:** Timeseries forecasting, Analytic Modeling, Ensembles, Small Data, Coronavirus COVID-19, Nigeria NCDC

## Abstract

The novel Coronavirus disease (COVID-19) was first identified in Wuhan, China in December 2019 but later spread to other parts of the world. The disease as at the point of writing this paper has been declared a pandemic by the World Health Organization (WHO). The application of mathematical models, artificial intelligence, big data, and similar methodologies are potential tools to predict the extent of the spread and effectiveness of containment strategies to stem the transmission of this disease. In societies with constrained data infrastructures, modeling and forecasting COVID-19 becomes an extremely difficult endeavor. Nonetheless, we propose an online forecasting mechanism that streams data from the Nigeria Center for Disease Control to update the parameters of an ensemble model which in turn provides updated COVID-19 forecasts every 24 hours. The ensemble combines an Auto-Regressive Integrated Moving Average model (ARIMA), Prophet - an additive regression model developed by Facebook, and a Holt-Winters Exponential Smoothing model combined with Generalized Autoregressive Conditional Heteroscedasticity (GARCH). The outcomes of these efforts are expected to provide academic thrust in guiding the policymakers in the deployment of containment strategies and/or assessment of containment interventions in stemming the spread of the disease in Nigeria

Specifications Table

**Table:** 

Subject	Decision Sciences
Specific subject area	Application of an online framework for forecasting the range of COVID-19 cases in Nigeria using limited data.
Type of data	Table Image Graph Figure
How data were acquired	Nigeria Center for Disease Control – NCDC http://covid19.ncdc.gov.ng/ COVID-19 Pandemic in Nigeria – Wikipedia http://tiny.cc/nigeria_covid19
Data format	The dataset is in raw Excel file format, has been cleaned, analyzed in this article, and is available in the Mendeley Data repository.
Parameters for data collection	The ensemble algorithm presents lower and upper bounds for the total number of possible COVID-19 cases for the next day. ARIMA, Prophet, and Holt-Winters models were implemented and tuned using brute search.
Description of data collection	Data on the daily number of COVID-19 cases in Nigeria were automatically mined from the NCDC and Wikipedia websites every 24 hours. The dataset contains COVID-19 cases starting from February 27, 2020, to April 5, 2020. Descriptive analysis of the dataset has been presented and the forecasts using limited data are made available. Sample code to mine real-time COVID-19 cases in Nigeria as a Pandas data frame or Excel file has been provided in the repository.
Data source location	Osun State University, Osogbo, Osun State, Nigeria.
Data accessibility	The data at the point of writing and code can be found in the Mendeley Repository. Repository name: Mendeley Data identification number: NigeriaCOVID-19 Data Repository: http://dx.doi.org/10.17632/3xsb9nyfhp.1 Project URL: https://github.com/kbmajeed/nigeria_covid19

## Value of the data

• These data are useful as they present facts that drive analytics on COVID-19 cases in Nigeria.
• Academic institutions, public health agencies, scientific communities, researchers, students, and self-explorers can use these data, code, and models to analyze COVID-19 cases in Nigeria and beyond.
• The data – with daily updates, model, code, and analysis presented can be applied to drive analytics, policy development, and decision making in other countries where data is scarce. It also represents an early reference that can be used in the future.
• The ensemble of models leverages the strengths and compensates for weaknesses in the individual forecasting algorithms even with limited data.

## 1 Data Description

**Fig. 1:**
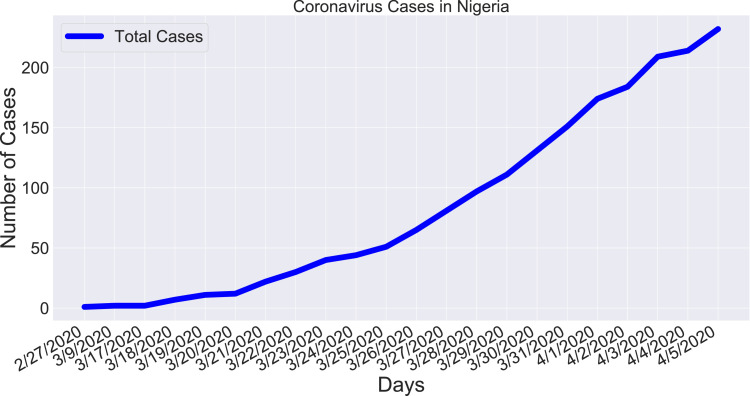
COVID-19 cases in Nigeria from March 2020 to April 5, 2020.

**Fig. 2:**
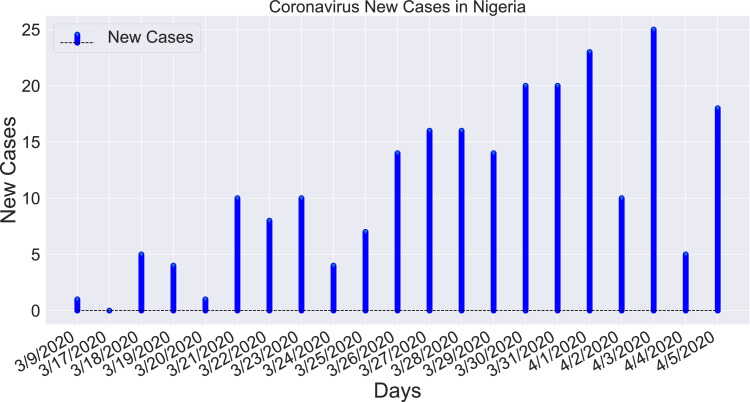
COVID-19 new cases in Nigeria from March 2020 to April 5, 2020.

**Fig. 3:**
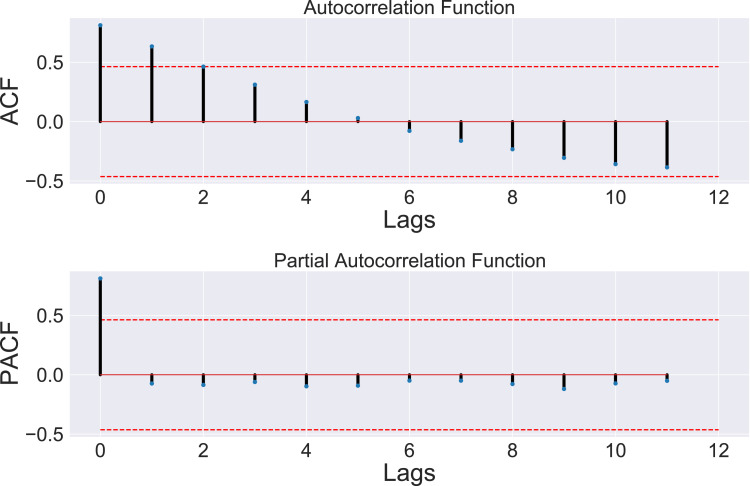
Autocorrelation (top) and Partial Correlation (bottom) plots of the *NigeriaCovid-19* dataset.

**Fig. 4:**
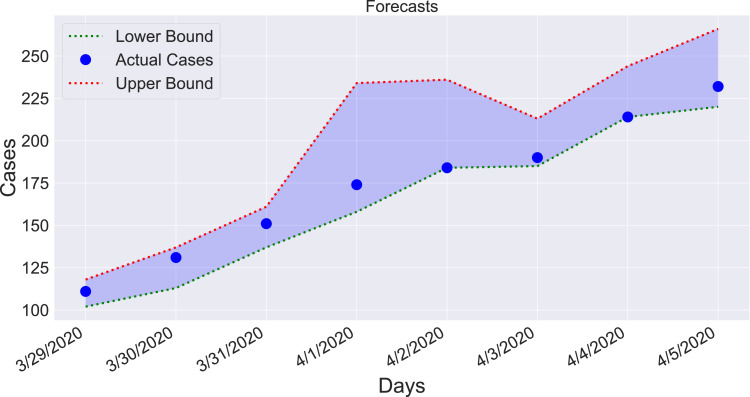
Forecasted region versus actual cases reported by NCDC.

**Fig. 5:**
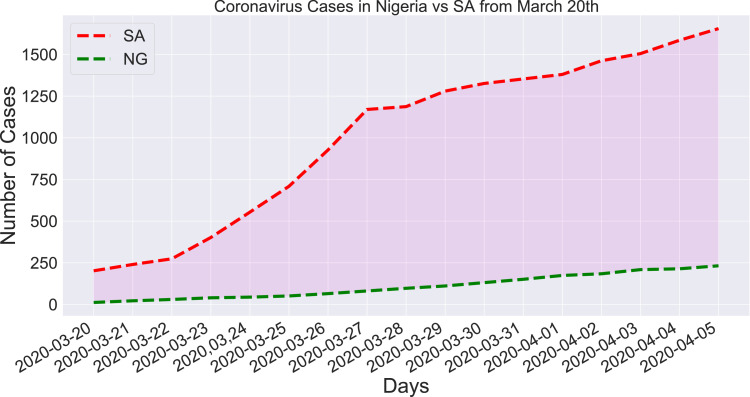
Nigeria vs South Africa Comparison from 20^th^ of March.

The daily number of COVID-19 cases in Nigeria from February 27, 2020, to April 5, 2020, were automatically mined every 24 hours from the official websites of NCDC (http://covid19.ncdc.gov.ng/) and Wikipedia (http://tiny.cc/nigeria_covid19) using a python script. The case numbers up to April 5 can be found in the supplemental data (Appendix A). As at the point of writing this brief, the dataset contains 39 time-series data points. For forecasting purposes, earlier days with zero incidences of COVID-19 were filtered for building the forecast models. This is presented in tabular form in Table 1-2 and visual form in Fig. 1 while the number of new cases per day is shown in Fig. 2. Seven successive daily forecasts by the ensemble, starting from March 29, 2020, to April 5, 2020, are presented in Table 3. The autocorrelation and partial autocorrelation behavior of the dataset of Fig. 3 aids in developing ARIMA modeling insights. Similarly, Fig. 4 is a visualization of the forecast beams indicating the direction and strength of increases or decreases in the forecasted number of cases. Table 4 highlights the relative strength and weaknesses of the models individually and finally; Fig. 5 presents a visual comparison of COVID-19 cases in Nigeria vs South Africa for analyzing policy impact.

**Table 1:** Description of the *NigeriaCOVID-19* dataset.

S/N	Property	Value
1	Description	Timeseries data
2	[Min, Max]	[0, 232]
3	Characteristics	Strong trend
4	Behavior	Non-stationary
5	Start date	February 27, 2020
6	End date	April 5, 2020
7	Outliers, Missing	None

**Table 2:** Percentage change in COVID-19 cases in Nigeria (Filtered)

S/N	Date	Total Cases	New Cases	Percent Change %
1	2020-02-27	1	1	(n.a.)
2	2020-03-09	2	1	+100
3	2020-03-17	2	1	+100
4	2020-03-18	7	5	+250
5	2020-03-19	11	4	+57
6	2020-03-20	12	1	+9
7	2020-03-21	22	10	+91
8	2020-03-22	30	8	+36
9	2020-03-23	40	10	+33
10	2020-03-24	44	4	+10
11	2020-03-25	51	7	+16
12	2020-03-26	65	14	+27
13	2020-03-27	81	16	+25
14	2020-03-28	97	16	+20
15	2020-03-29	111	14	+14
16	2020-03-30	131	20	+18
17	2020-03-31	151^1^	20	+15
18	2020-04-01	174	23	+15
19	2020-04-02	184	10	+6
20	2020-04-03	209^2^	26	+14
21	2020-04-04	214	5	+2
22	2020-04-05	232	18	+5

1 On the 31st of March 2020, the NCDC reported 139 on twitter while Wikipedia updated its 139 cases to 151 later by midnight.

2 On the 3rd of April 2020, the NCDC reported 210 cases. This was later corrected to 209 cases the next day.

**Table 3:** COVID-19 Forecasted cases (lower and upper bounds) and Official NCDC cases.

S/N	Forecasted Day	Forecasted Cases	Official Cases	Within Range?
1	2020-03-29	[102 - 118]	111	Yes
2	2020-03-30	[113 - 137]	131	Yes
3	2020-03-31	[137 - 161]	151	Yes
4	2020-04-01	[158 - 234]	174	Yes
5	2020-04-02	[184 - 236]	184	Yes
6	2020-04-03	[185 - 213]	209	Yes
7	2020-04-04	[214 - 244]	214	Yes
8	2020-04-05	[220 - 266]	232	Yes

**Table 4:** Some Strengths and Weaknesses of the Individual Models.

Model	Strengths	Weaknesses
**ARIMA**	Few parameters, interpretable, short-term forecasts	Requires stationary data, unusual trends.
**Prophet**	Outliers, Missing data, Speed, Robust, Powerful, Strong seasonal effects, Long forecasting, Automatic.	No readily known weaknesses
**Holt-Winters ES**	Strong and accurate forecasting (short-term), favors recent data samples, requires few data points, straightforward implementation.	Lagged forecasts.

## 2 Experimental Design, Materials, and Methods

In this paper, we present the application of ensemble forecasting models in a data constrained environment. The objective is to establish the lower and upper bounds on the possible number of COVID-19 cases per day using a framework that automatically streams web data in real-time from reliable sources. This data is used for retraining and adapting the parameters of an ensemble of three models which in turn updates its forecast prediction for the following day. Each of the three models provides an estimated lower bound and upper bound for the number of cases. The Ensemble forecast is achieved by taking the minimum of the lower bounds and the maximum of the upper bounds. Due to limited sources of data (the only available information is a single variable, “number of COVID-19 cases per day”), it makes it difficult to implement specialized, advanced, and more generalizable methods that often require a variety of features and bigger datasets. It is also important to highlight that there are only a few samples (22) in the dataset making it equally challenging to apply nonparametric neural models.

The Nigeria Center for Disease Control (NCDC) records the number of cases of COVID-19 in Nigeria using established epidemiological methods [1]. This data is presented to the public on social media several times a day as updates arrive and are available at NCDC secretariat/website. Unfortunately, information about the number of tests carried out per day and other factors are not readily available. This leaves us with only one variable: the total number of cases. In the same vein, Wikipedia maintains a data table of the number of COVID-19 cases in Nigeria with values updated using information from NCDC as well as other reliable verified news and media outlets. Surprisingly, even under data and information constraints, bounds on our ensemble forecast has been able to accurately capture the daily total number of cases from March 29, 2020, to April 5, 2020 (Tables 1 and 2; Figs. 1 and 2). We also provide an informative data visualization comparing COVID-19 cases in South Africa after policy impact.

## 3 Ensemble of Forecasting Algorithms

**i. Autoregressive Integrated Moving Average** (ARIMA) is made up of integrated Autoregressive and Moving Average components and has the form ARIMA (p, d, q):

$$y_{t}^{\prime }=c+\phi_{1}y_{t-1}^{\prime }+\ldots +\;\phi_{p}y_{t-p}^{\prime }+\theta_{1}\varepsilon_{t-1}+\ldots +\theta_{q}\varepsilon_{t-q}+\varepsilon_{t}$$

$y_{t}^{\prime }$ represents the series of differences, *ϕ_i_* are the coefficients, the $y_{t-p}$ and $\varepsilon_{t-q}$ are the lagged predictors for the model [2]. The ARIMA model is a generalization of many sub-models and characterized by three parameters: order of autoregressive observations *p*, degree of differencing *d*, and number of moving average terms *q*. The foundations of ARIMA lie on the fact that nonstationary time-series can be made stationary by through differencing. The verification of stationarity can be achieved using autocorrelation plots and unit root tests such as the Kwiatkowski-Phillips-Schmidt-Shin (KPSS) test [3]. For this model, we are interested in non-seasonal phenomena. Using brute search and inspection of the autocorrelation function ACF and partial autocorrelation function PACF plots (Fig. 3), an ARIMA (2,1,0) model was chosen.

**ii. Prophet** is an additive regression time-series forecasting algorithm developed by Facebook [4,5]. It has strengths in dealing with strong seasonal effects, missing data, outliers, and shifts in trend making it fully automatic. It is also implemented with a Stan backend which introduces a fast solution to L-BFGS (Limited-memory Broyden-Fletcher-Goldfarb-Shannon algorithm) for the forecasting problem. *Prophet* includes a decomposable time series model defined by:

y(t)=g(t)+s(t)+h(t)+ε(t)

*g*(t) is the trend, *s*(t) represents seasonal changes and *h*(*t*) captures irregular effects. We elicit trend characteristics *g*(*t*) as of primary importance in developing the forecast. The trend model in this work utilizes a piecewise saturated growth model with time-varying carrying capacity defined below:

$$g\left(t\right)=\frac{C\left(t\right)}{1+\text{exp}\left(-\left(k+\alpha {\left(t\right)}^{⊺}\delta \right)\left(t-\left(m+\alpha {\left(t\right)}^{⊺}\gamma \right)\right)\right)}$$

*C*(*t*) is the time-varying carrying capacity, *k* is the growth rate, *m* is an offset. The growth rate is not constant but piecewise with *α*(*t*), *γ*, and *δ* defining its structure.

**iii. Holt-Winters Exponential smoothing** is a famous time-series modeling and forecasting algorithm that came to light in the 1950s after some scientific reports [6], [7], [8]. This algorithm is a weighted average of past observations with exponentially decaying weights to capture the trend in a time-series dataset. It has the general form of:

$${\hat{y}}_{T+1|T}=\alpha y_{T}+\alpha \left(1-\alpha \right)y_{T-1}+\alpha {\left(1-\alpha \right)}^{2}y_{T-2}+\ldots$$

Where the values of alpha tune the response of the model. Alpha values close to 0 emphasize past input data, while values close to 1 emphasize recent input data. The Holt-Winters ES algorithm introduces a seasonality component to the vanilla ES. In addition to the ES model, a GARCH model [9] was used to forecast variances and combined with the ES model gives the upper and lower bound for this model

**iv. Forecasts:** In Table 3, the dates for each of the forecasts along with the corresponding acutal reported (official) cases by NCDC can be observed. The performance of the ensemble models can easily be visualized in Fig. 4. The forecast envelope accurately captures the actual number of real cases detected by the NCDC.

By combining these three algorithms, we compensate for their relative weaknesses while reinforcing their relative strengths (Table 4). Ultimately, we believe in the mantra that All models are wrong, but some are useful – George Box.

## 4 Visualization of data comparing COVID-19 cases in South Africa with Nigeria and policy impact

Although, there is more information on the COVID-19 situation in North America, Europe, and Asia in comparison with Nigeria. Fig. 5 presents the comparison of COVID 19 cases in South Africa (SA) with Nigeria (NG). The figure shows that the number of confirmed cases of the infection in SA in geometrically higher than NG. This observation could be explained from two perspectives: (i) SA either has much more people infected with COVID-19 than NG; (ii) or there is wider coverage in the number of people test per day among the population. The latter reason may probably be stronger than the former as there is corroboratory evidence for the later. As of the 20th March 2020, SA had conducted 6,438 tests [10] while Nigeria only performed 69 tests [11].

One of the current strategies being deployed for the containment of COVID-19 is the lockdown of regions affected to avoid further spread by human movement. SA declared a National lockdown on the 26th of March, 2020 [12] to tame the spread of infection. As shown in Fig. 5, there was a kink in the progression of confirmed cases on March 27, 2020. The steepness of the curve was relatively flattened and steady for a few days thereafter. The steep upward trend of the infections was stemmed the same day the national lockdown policy was announced. The fact that those that have been infected before the lockdown will be presenting themselves for tests and treatment could have accounted for a slight rise in cases within 2 weeks of lockdown. The full compliance with the policy and number of new cases in post-2-weeks lockdown policy are scenarios that would guide further actions in SA.

It is difficult to model COVID-19 (in fact, any real-life scenario) has inherent modeling difficulties such as the number of tests, randomness, interventions, stay-at-home compliance, curfews, epidemiological realities, and many other factors contribute to the difficulty of forecast models in this case. Countries, especially in Africa who are just witnessing a progressive rise in COVID-19 cases must be decisive in implementing the containment interventions and ensure strict compliance by the citizenry.

## Appendix Supplementary materials

Image, application 1

Image, application 2
